# Building a profile of subjective well-being for social media users

**DOI:** 10.1371/journal.pone.0187278

**Published:** 2017-11-14

**Authors:** Lushi Chen, Tao Gong, Michal Kosinski, David Stillwell, Robert L. Davidson

**Affiliations:** 1 Institute for Language, Cognition and Computation, School of Informatics, University of Edinburgh, Edinburgh, United Kingdom; 2 Haskins Laboratories, New Haven, Connecticut, United States of America; 3 Center for Linguistics and Applied Linguistics, Guangdong University of Foreign Studies, Guangzhou, China; 4 Graduate School of Business, Stanford University, Stanford, California, United States of America; 5 Judge Business School, University of Cambridge, Cambridge, United Kingdom; 6 Two Cloaks Ltd., Glasgow, United Kingdom; 7 Scientists for EU Ltd., London, United Kingdom; Dalian University of Technology, CHINA

## Abstract

Subjective well-being includes ‘affect’ and ‘satisfaction with life’ (SWL). This study proposes a unified approach to construct a profile of subjective well-being based on social media language in Facebook status updates. We apply sentiment analysis to generate users’ affect scores, and train a random forest model to predict SWL using affect scores and other language features of the status updates. Results show that: the computer-selected features resemble the key predictors of SWL as identified in early studies; the machine-predicted SWL is moderately correlated with the self-reported SWL (*r* = 0.36, *p* < 0.01), indicating that language-based assessment can constitute valid SWL measures; the machine-assessed affect scores resemble those reported in a previous experimental study; and the machine-predicted subjective well-being profile can also reflect other psychological traits like depression (*r* = 0.24, *p* < 0.01). This study provides important insights for psychological prediction using multiple, machine-assessed components and longitudinal or dense psychological assessment using social media language.

## Introduction

Subjective well-being (SWB) is a broad concept referring to individuals’ cognitive and affective evaluations of their lives. The cognitive evaluation is called satisfaction with life (SWL), which is self-assessment of one’s own life. The affective component is what we usually refer to as “happiness”. It is an aggregation of a person’s emotion and mood within a period of time [1]. These two domains have been widely studied due to their interactive effects on our mental health [2,3].

SWL is often evaluated by a five-item scale, which assesses individual’s memory-based life experience based on a series of questions [4]. An example question is “Are you satisfied with your life?” Such memory-based assessment tends to neglect experience duration, and shows bias towards peak or end experience. Therefore, one time self-reported SWL is subject to distortion of impression management [1,5,6]. A person may report low SWL after a recent break-up with a partner, even though many positive events have occurred since the break-up; in other words, the ‘peak experience’ (break-up) distorts appraisal of more recent events [5]. In addition to cognitive bias, SWL is also subject to a set of internal and external criteria. The internal criteria include individuals’ mental structures for organizing and interpreting information, and the externally imposed criteria involve a person’s current circumstances such as health, income, and location [7].

SWL is a relatively stable variable. The test-retest reliability of SWL is 0.84 in two months, and 0.54 in four years [5]. By contrast, affect is less stable. Affect is a neurophysiological state consisting of hedonic (pleasure-displeasure) and arousal values [8]. It can be measured by a single-occasion Positive and Negative Affect Schedule (PANAS) [9]. Participants are asked to rate how they feel right now, today, during the past week, during the past few weeks, and during the past year. The test-retest reliability ranges from 0.54 to 0.68. When participants are asked to recall affect from more distant time points, the responses could be repeatable [10], because the responses are guided more by stable traits such as cognitive bias and impression management than by better recollection of affect. Noting this, psychologists have developed a compensatory approach—experience sampling. Following this approach, participants are asked to report their moods several times a day at randomly-selected time points [10,11,12]. Since it consists of a series of short-term reports [10], the experience sampling is also referred to as moment-based mood report. In addition to the moment-based intensity of affect, it has been argued that the basis of affective well-being is formed by the total amount of time during which a person experiences pleasant emotions versus unpleasant ones [13]. The relative frequency of positive affect is highly linked to self-report well-being measures. Nonetheless, a major limitation of experience sampling is that it is resource-consuming and very demanding for participants. Therefore, it is difficult or nearly possible to conduct a longitudinal study using this approach.

Recently, researchers have started to make use of social media and digital databases to complement self-reporting. Social media websites contain large corpora of language data, which may reflect social relations [14], emotions, and life events [15] of the users. Based on computational approaches, we can utilize social media data to predict users’ general happiness [16,17,18,19]. For example, one study attempts to predict users’ happiness based on their behaviors on dozens of Facebook functions (e.g., status updates and number of “likes”) [20]. In another study, a word counting approach is adopted to estimate the SWB of the people living 200 years ago based on the language corpora derived from millions of Google Books from six countries [21].

**Fig 1:**
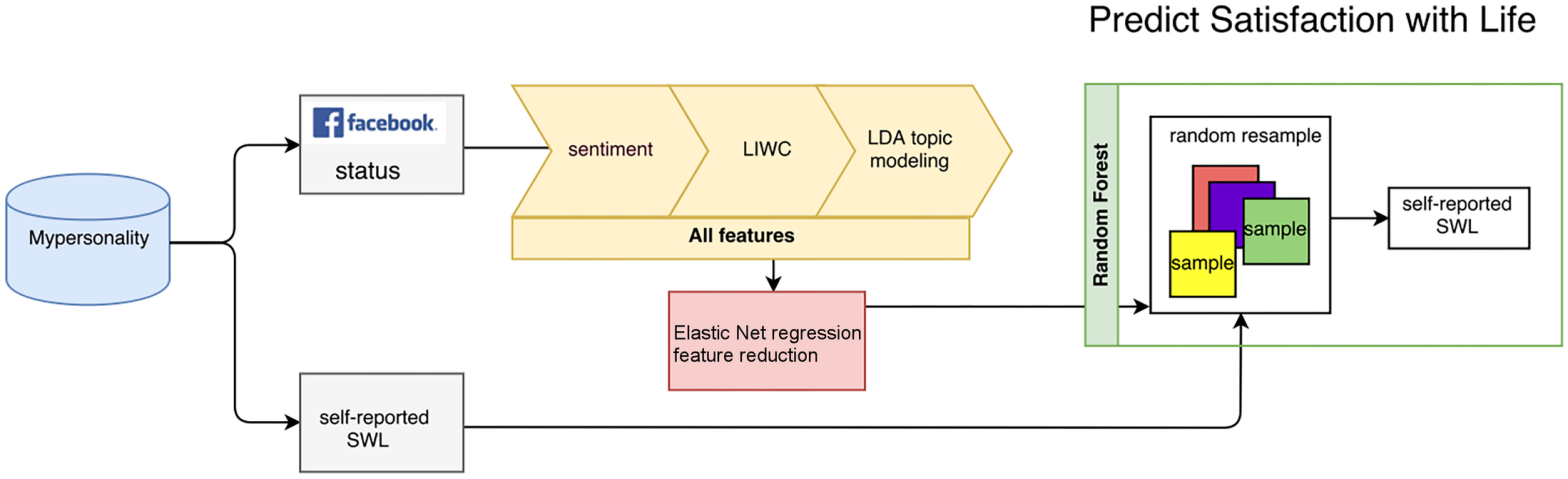
Satisfaction with life prediction model. We use Elastic Net regression to select informative features among sentiment, LIWC and LDA generated topics for the random forest model. The model is trained to fit the self-reported SWL score.

Many of such “Big Data” models usually predict the variable as a whole [22], whereas the predicted variable consists of several constructs. For instance, SWB consists of affect [16,17,18] and SWL [23], and affect should be measured by both intensity and frequency [13]. Noting this, in this paper, we adopt a unified approach to predict the major constructs of SWB using the status updates provided by the myPersonality project (http://www.mypersonality.org/), and combine the machine-predicted constructs into a SWB profile. We first use sentiment analysis to infer users’ affect from their Facebook status updates, and break this affective construct into intensity and frequency. We then combine affect with other features to train a machine learning model to predict SWL (Fig 1), and estimate prediction performance based on correlation between the machine predicted SWL and self-reported SWL.

To verify predicted affect based on sentiment analysis, we investigate users’ affect scores when they mention different activities, and compare our results with those in a previous experimental study based on moment-based mood reports [10]. We also evaluate the predictive performance of our SWB profile consisting of predicted affect and SWL scores by correlating the SWB profile with depression symptoms. Previous studies show that both affect and SWL are significant predictors of depression [24,25]. An SWB profile containing multiple machine-predicted constructs leads to a better prediction of depression symptoms than a SWB profile having a single construct.

## Methods and materials

### Data and participants

Our study uses the Facebook status updates from individuals who participated in the myPersonality project from January 2009 to December 2011 [26]. The protocol of our study was approved by myPersonality. The methods were carried out in accordance with the approved guidelines from myPersonality. myPersonality is a Facebook-based application providing psychometric tests and corresponding feedbacks to its users. Some of the participants allowed myPersonality to collect their account ‘profiles’ and public ‘status updates’ in Facebook. Currently, myPersonality has over 7.5 million users, of whom 40% have allowed data retrieval. Data collection of myPersonality complied with the terms of Facebook service. All data are anonymized and gathered with opt-in consents for research purposes.

The sample used in our study contains all the participants (*n* = 3324) who have not only completed the SWL scale between the years 2009 and 2011 but also allowed myPersonality to collect their Facebook status updates. The number of participants is reduced to 2612 after we filter out users having a small number of status updates (total number of updates < 30). The mean SWL score of this population is 4.2, similar to the estimate of a general population produced by a prominent meta-analysis [5]. The SWL scores were measured using a five-item SWL scale [4], which is suitable for a wide range of age groups and shows good validity and reliability. For the main findings reported in this paper, we select the Facebook status updates covering the full time-range (2009 to 2011) to build the SWL model, since life satisfaction assessed by the five-item SWL scale remains relatively stable over a period of up to four years [27]. In S1 Text, we show the outcomes of repeating the same analysis on the data within the 30 days prior to the SWL survey for each user. These are not included in the main text, because of the reduced sizes of the data. All analyses are carried out based on the functions in related packages of R 3.2.4 [28].

Data Availability: All result files are available at https://github.com/gtojty/FB_ML. The result files that we made available on the GitHub repository contain the data necessary to reproduce the Tables and Figures contained in the document. The authors are not authorized, however, to share the individual-level Facebook data because it would with be an IRB ethics violation—the privacy of participants would be compromised. Interested users with appropriate CITI certification and IRB approval can contact the MyPersonality Application (http://mypersonality.org/wiki/doku.php?id=database_use_guidelines) for permission to access the original dataset. This sensitive and private data is not available directly through the Facebook API.

### Data preprocessing

After filtering out the users having few posts (< 30), we clean the remaining status update texts using regular expressions. First, hyperlinks and digits are removed. “Smileys”, usually formed by punctuations, are another type of text-based valence indicator commonly used in social media text [29]. We convert the “smileys” into words for ease of interpretation and further cleaning of the data. For instance, “:-)” is replaced by “happyface”, and “:-(”by “sadface”, etc. Conversion of smileys allows for collation of multiple punctuation patterns to a single concept, e.g. “:)” can be considered as the same ‘happyface’ concept. It also accounts for the common practice of explicit writing, e.g. ‘happyface’, as a long-form of the punctuation pattern. Finally, we remove the other punctuations. In many natural language processing pipelines, it is a common practice of removing ‘stop words’, e.g. “a”, “am”, “and”, etc. In our study, we use pre-defined word and topic lists that have already taken account of stop words.

### Feature extraction

#### Affect

A common approach to evaluate affect is sentiment analysis, which is a family of analyses using word valence to classify the opinion and mood of the text [29,30,31]. Valence is the foundation of affect, e.g. fear, anger, etc. People’s affect is aroused by the invariant valence of an event or object. One of the basic tasks in sentiment analysis is polarity detection, which is a binary classification task with outputs as ‘positive’ or ‘negative’ [31]. In this study, we adopt the polarity detection approach. We use a simple computer algorithm to rate the valence of each status update according to a predefined list of valence words [29,32,33]. A positive valence word adds +1 to the valence word count, whereas a negative valence word adds -1 to the valence word count. The performance of sentiment analysis is dependent on the list of words used. We initially use a published list derived from news reporting (6800 words) [34], but social media language is quite distinct from news reporting in style. We then augment this list with the words representing the smileys (e.g. happyface, sadface) and other valence-related words commonly found in Facebook status updates. Finally, the approach of valence word count is used to generate a sentiment score for each status, which is the sum of valence words divided by the number of status updates of each user. Here, we normalize by the total number of status updates rather than the number of words in the relevant status text, due to the uneven numbers of words in Facebook status updates. For example, 50115 status updates contain fewer than four words, 115047 status updates contain more than 20 words, and the other 308007 updates contain 4 to 20 words. The total number of status updates is therefore used as an estimate of how many words a user expresses on Facebook. We then calculate the mean status sentiment score of each user and use it as a feature in the prediction model.

The relative frequencies of positive and negative status updates are computed as overall positive count divided by the total count of a user and overall negative count divided by the total count of a user. Considering that the basis of affective well-being is formed by the total amount of time during which a person experiences pleasant emotions versus unpleasant ones [27], we create a ratio of status updates for each user (overall positive count divided by overall negative count). In sum, the affective features include: the intensity of affect (mean sentiment score), the frequency of affect (frequency of positive status, frequency of negative status), and the ratio of positive status to negative status.

#### Topic clusters

Creating topics in a text is a common approach to classify the text. In this study, we combine manually created topics and machine created topics as features for the prediction model. Pennebaker and colleagues defined 66 psychologically meaningful categories called Linguistic Inquiry and Word Count (LIWC) [35,36], which have been used to classify text and predict behavior outcomes. Using pre-defined lists and categories, LIWC is also referred to as the “closed-vocabulary” approach.

A more advanced approach to classify a text according to topics is to generate the topics base on the data for analysis, which is also called the “open-vocabulary” approach. For example, Latent Dirichlet Allocation (LDA) [37] uses the observed corpus to produce a probabilistic relationship between words, ‘topics’ and documents. The user assumes the number of topics within the corpus, and the solution provides a matrix of probabilities, each item in which records the probability with which each document includes each topic.

The number of documents is of paramount importance in LDA. For example, one study based on Twitter data (similar in properties to Facebook status updates) finds that the LDA performance stabilizes with at least 4000 documents, and that the larger the number of documents, the more accurate the LDA topic results [38]. In our study, we consider the sample of 2612 participants as insufficient for a robust topic creation. Therefore, we borrow the LDA topics generated from 75000 Facebook volunteers [39]. There are in total 2000 topics, with 20 or fewer words ‘within’ each topic (a threshold has been applied to the probabilities that link words to topics).

A recent study uses these document-topic probabilities as features to predict SWL, but it only achieves a correlation of 0.24 with the self-reported SWL [40]. We believe that while the topic lists are meaningful and useful, the truncated topics can produce odd effects in a dataset, especially for users having small total word counts. The calculation of the probability that a topic is referred to by a user is:

$$p\left(t|u\right)=\Sigma_{w\in words_{u}}p\left(t|w\right)*p\left(w|u\right)$$ (1)

where *p(t|w)* is the probability of a topic, given a word (calculated from the truncated frequency table) and *p(w|u)* is the user’s probability of mentioning a word. Under normal circumstances, every word produces a score for every topic in every user. Since the table has been truncated, most words do not produce a score for each topic. Also, *p(t|w)* can be higher than it should be. For example, the word “driveway” only appears in the topic referring to snow clearance for traffic in winter, thus *p(t|w)* = 1 in the published table, whereas the word “ice” has *p(t|w)* = 0.049 for the same topic. This overestimates the topic relevance of “driveway” while relatively underestimating “ice.” At the same time, *p(w|u)* is increased when a user has a relatively small total word count, which potentially inflates the importance of words for some users, especially in social media data where users’ word counts vary greatly. The end result is an artificial increase in the ratio between high and low *p(t|u)*.

Noting this, we adopt a word count approach for the published LDA topic words. For each user, we concatenate all his/her status update texts into one ‘document’. We then compute the document word frequency for all the topic words and sum up the word frequency in each topic to form a topic frequency score. A topic frequency score shows how often a document overlaps with the topic. The matrix of all topic frequencies of every user is used as a feature in the prediction model (after Elastic Net regularization is used for feature reduction, see the next section).

This way of scoring has a different impact on users’ score for each topic. Use of a word increases the user’s score for every topic list containing the word by 1, regardless of *p(t|u)*. If a user mentions “driveway”, we increase his/her score for the winter traffic topic by 1, but keep the same score for the use of the word “ice.” There are 1147 words that appear only in one topic in the truncated table, where *p(t|w)* = 1. The word count approach reduces the artificial importance of these words. At the same time, a user would be given the same score increase of 1 in all six topics that contain the word “ice.” This may conversely inflate the association between the user and some of the topics. Yet, since status updates are often very brief, there could be a benefit by assuming that a user will not use many topic words even when talking about a topic. Our word count approach follows this assumption. Using this approach, the ratio between the highest and lowest topic scores (where score > 0) for any user is reduced, compared to that using the probability formula. This provides a more flexible estimation of user-topic relationship.

#### Topic reduction

Too many sparse features can pose problems for machine learning algorithms and not all topic clusters are equally informative for predicting SWL. We thus need to reduce the number of topics clusters to be fed into the prediction model. We use Elastic Net regression [41] for this purpose. Elastic Net is a regression method that combines the penalizing factors from both Ridge and LASSO (Least Absolute Shrinkage and Selection Operator) regression methods [42]. It improves upon LASSO regression by allowing for correlated variables in the model, whereas LASSO is prone to removing all but one variable in a correlated set. Upon applying such method, some of the regression coefficients may be reduced to zero, which may be removed from the model. To minimize the sparsity of the result, we choose a small penalty (alpha = 0.1).

To avoid over-fitting over the course of the full pipeline, we split the original sample into a training sample (*n* = 1873), used for the Elastic Net regression and random forest model building, and a testing sample (*n* = 739), used to evaluate the prediction performance of the whole pipeline.

#### SWL prediction model

Previous studies often use statistical regression algorithms to build prediction models. Such algorithms assume that the dataset under study can be summarized by a single probability distribution. This may lead to high biases. Alternatively, the algorithms with high-variance profiles such as decision tree or supporting vector machine can generate arbitrarily complex models to better fit data variation [43]. In this study, we choose the random forest as the learning algorithm for the SWL model.

Random forest is an efficient algorithm based on an ‘aggregation’ idea [44]. It is simply a collection of decision trees whose results are aggregated into one final result. Its principle is to build multiple binary decision trees by bootstrapping samples randomly selected from the training dataset. Each tree is trained on a randomly selected sample of the training data, and the predictions are made by the majority vote of these trees. In this way, random forest can minimize errors due to bias and variance.

We conduct a manual search on the number of trees to grow (ntree) and the number of variables randomly sampled as candidates at each split (ntry), by creating eight models each having different sets of parameters (ntree = 500, 1000, 1500, or 2000; and ntry = 2 or 3). Among these models, the one with ntree = 1000 and ntry = 3 generates the best matching results and thus is reported here. Since bootstrapping uses a random sample of the data, there is also a subset of the training data not used during training. This set can be applied to internal validation, namely the ‘out-of-bag’ (OOB) performance estimate (see the formulas in [45]). In our study, about 37% of the training instances end up being out-of-bag in each round. The self-reported SWL scores serve as the target for training.

The trained model is used to predict SWL for the independent test dataset (*n* = 739) as a performance estimate for the entire process. We use Pearson correlation to compare the machine-predicted with the self-reported SWL scores, and report root mean square error (RMSE). As a baseline, we report the performance of a ‘naïve’ prediction using solely the median value of the self-reported SWL scores as the feature in the prediction model. To calculate correlation statistics, we add a small distortion (a variable randomly chosen from a uniform distribution between 0 and 0.001) to this median value.

#### Predictive performance of SWL model

Predictive performance measures the extent to which a score on a test predicts a score based on other measures (e.g., to what extent SWL scores correlate with the screening test for depression and depressive disorder). myPersonality provided dozens of measurement scales for users to complete, and motivated participation by feedbacks about the scores obtained. Some participants completed many scales but uptake was varied. Among the 2612 users who completed the SWL scale, some (*n* = 386) also completed a CES-D scale on depression symptoms [46]. We use the CES-D scale as the criteria for predictive performance of our SWB profile.

To use machine predicted SWL as an input feature in this process, we train a new random forest model on all 2612 users, using the best model parameters found in the process described above and accepting the out-of-bag prediction for SWL. The out-of-bag estimate is as accurate as the one obtained by using a test set of the same size as the training set [45].

We then evaluate how well each constituent of the machine predicted SWB profile (machine-predicted SWL and sentiment features) and the self-reported SWL share information with depression symptoms. This is achieved by calculating the correlations between these criterion variables and the CES-D test scores [18].

Evaluation of the predictive performance of the SWB profile is conducted by random forest. We build models using three sets of features: 1) sentiment (mean sentiment, frequencies of negative and positive status updates); 2) self-reported SWL and sentiment; 3) machine-predicted SWL and sentiment (the SWB profile). We divide the data into a training set (70%, n = 260) and a testing set (30%, n = 126), and predict self-reported CES-D scores using random forest. Performance of the prediction is evaluated by correlating the predicted CES-D values with the self-reported CES-D scores. We also report RMSE.

#### Activity sentiment score

In addition to the prediction models, we investigate the potential of using the Facebook language data as an alternative to ‘experience sampling’. A previous experience sampling study [10] finds that particular activities are associated with different degrees of happiness. For example, the affect scores of school activities are generally below the average affect score of all the activities, whereas the affect scores of social activities are rated above the average. In our study, we adapt the sentiment analysis described above to the status updates that also contain references to the activities described in that experience sampling study. We manually define a topic (set of words) for each activity (e.g. housework includes “vacuum”, “dishes”), and sum up the sentiment scores of the status updates that contain the selected terms (see S1 Table), hereafter ‘activity sentiment score’. The selected terms are rated independently by two researchers, and the inter-rater reliability is measured by Cohen’s Kappa (kappa = 0.36, *p* < 0.01). To compare with the experience sampling study, which shows *z*-scores calibrated on the mean self-reported affect score, we transform activity sentiment scores into *z*-scores, labeled as activity sentiments.

## Results

### Affect

Sentiment scores reflect the intensity of users’ affect. Among the total 473169 status updates from 2612 users, 184831 are identified as positive, 115915 as negative and 172423 as neutral. 2088 out of 2612 users have more positive status than negative status. Sentiment scores of the current sample indicate that people tend to show positive or neutral affect in their status updates. Users’ mean sentiment scores are moderately correlated with the self-reported SWL (*r* = 0.21, *p* < 0.01). In our study, the affect computed with the modified list (including smileys, etc.) has a similar correlation with the self-reported SWL as reported in the previous study [47], positive (*r* = 0.08, *p* < 0.01) and negative (*r* = -0.23, *p* < 0.01). Whereas, the affect computed with the original list [34] is less correlated with the self-reported SWL, positive (*r* = 0.04, *p* < 0.05) and negative (*r* = -0.22, *p* < 0.01). This indicates that the modified sentiment word list can better reflect users’ affect than the original list. In addition, the correlation matrix shows that the intensity of affect, frequencies of affect and proportion of positive affect are moderately to highly correlated with each other (see Table 1).

**Table 1:** Correlation matrix of affect features and self-reported SWL.

	1.	2.	3.	4.	5.
1.self-reported SWL					
2. sentiment	0.21				
3. positive frequency	0.08	0.68			
4. negative frequency	-0.23	-0.50	0.12		
5. positive / negative	0.16	0.68	0.45	-0.48	

All the results have *p* values less than 0.001.

Sentiment: mean sentiment score of a user; positive frequency: proportion of positive status among all status of a user; negative frequency: proportion of negative status among all status of a user; positive/negative: ratio between positive and negative frequency.

### Computer-selected features

The Elastic Net regression selects 117 topics from the 2000 LDA topics and 13 topics from the 66 LIWC topics. These topics include: school achievements (e.g., graduation), outdoor activities (e.g., fishing, skiing), play (e.g., game, fun), swear words, affirmative behaviors (e.g., promise, guarantee), entertainment (e.g., TV, music), negation (e.g., shouldn’t, couldn’t), baking, religious, positive or negative feeling, new born (e.g., baby, face), holiday, physical complaint, school achievement, and nightlife. In general, the topics selected by the Elastic Net regression resemble the internal and external SWL criteria as suggested in earlier studies, including: people’s mental structures for interpreting information [35] and current circumstances [1,2]. Physical complaint or school achievement topic reflects how people interpret things that have happened in their lives. Individuals using more negative feeling words tend to interpret things negatively, which often contributes to depression and anxiety. Other topics, such as outdoor activities, school achievements, new born, and so on, reflect individuals’ current circumstances. Notably, income, recognized as a major factor contributing to SWL, is not identified directly in the Facebook status updates, perhaps because explicitly referring to financial status is not considered socially appropriate in public fora like Facebook.

### SWL model

The primary judgment of the performance of our prediction model is the correlation between the self-reported results and the machine-predicted ones. For the independent test set (30% of the participants), the model using 13 selected LIWC as features has a correlation (*r*) of 0.29 with the self-reported SWL scores, the model using selected 117 LDA topic clusters and affect as features has a much stronger correlation with self-reported value (0.34), and the model combining all the three aspects of features produces a correlation of 0.36, which outperforms a recent study on the same dataset using a different pipeline for predicting SWL [40]. All these correlations are significant (no adjustment for multiple testing). We also report RMSE, which indicates an error range from 1.30 to 1.37 SWL units (where the min SWL score is 1.2 and the max is 6.8) (see Table 2).

**Table 2:** Correlations between the prediction performance of the random forest models using different features and self-reported SWL.

Feature set	*r*	*p* value	RMSE
Baseline (1)	0.001	0.97	1.37
LIWC (13)	0.29	1.3e ^-15^	1.32
selected LDA (117)	0.33	< 2.2e ^-16^	1.32
selected LDA + sentiment (120)	0.34	< 2.2e ^-16^	1.31
selected LDA + selected LIWC + sentiment (133)	0.36	< 2.2e ^-16^	1.30

The baseline model uses the median of the self-reported SWL with variation as feature. Root mean square error (RMSE) is relative to a range of SWL scores from the full dataset of 1.2 to 6.8. Numbers within brackets in the ‘feature set’ column are numbers of features in those sets.

We measure the variable importance inside the random forest model using the ‘varimp’ function of the Party library, which calculates the standard and conditional variable importance for ‘cforest’ using the principle of the ‘mean decrease in accuracy’ importance in random forest. S2 Table shows the variable importance results and S1 Fig shows the top 50 important variables in the random forest model.

### Predictive performance of subjective well-being profile

We find that both the self-reported SWL (*r* = -0.27, *p* < 0.01) and the machine-predicted SWL (*r* = -0.23, *p* < 0.01) are negatively correlated with the self-reported CES-D scores. The random forest prediction of CES-D indicates that SWB profile is able to boost this prediction of depression symptoms (see Table 3). Sentiment features alone produce a CES-D prediction that has mild but non-significant correlation with self-reported CES-D (r = 0.08, p = 0.381) Combining the machine-predicted SWL and sentiment features can predict depression symptoms (*r* = 0.25, *p* = 0.005), and combining the self-reported SWL and affect can generate more significant results (*r* = 0.28, *p* = 0.001). RMSE, reported in Table 3, is roughly 8 for each prediction, from a CES-D range of -20 to 80. This indicates that machine predicted SWL is a useful predictor of CES-D and although the affect/sentiment specific features do not boost the performance greatly, there may be scope for developing language feature variables for this purpose.

**Table 3:** Correlations between the random forest predicted and self-reported CES-D.

Feature set	*r*	*p* value	RMSE
Baseline (1)	-0.02	0.689	9.15
sentiment (3)	0.08	0.381	8.45
self-reported SWL + sentiment (4)	0.28	0.001	7.90
machine-predicted SWL + sentiment (4)	0.25	0.005	7.96

The baseline model uses the median of the self-reported SWL with variation as feature. Significance of the correlation (p value) and root mean squared error (RMSE) is also provided. Numbers within brackets in the ‘feature set’ column are numbers of features in those sets.

### Activity sentiment

**Fig 2:**
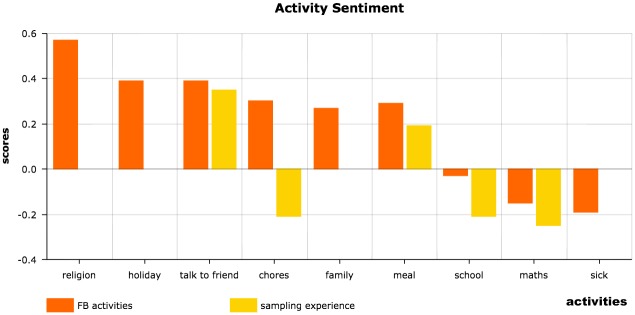
Activity sentiment scores. We compare the Facebook activity sentiment scores (FB activities (z-scores)) with the activity sentiment scores in the experience sampling study [11] (experience sampling (z-scores)). Since some activities were not included in the experience sampling study, the corresponding columns are empty.

Based on the language in Facebook status updates, our study finds that people tend to have higher sentiment scores when mentioning meals, chores, holidays, religion, whereas they have low scores when talking about death or diseases, mathematics and school on Facebook (see Fig 2). These findings show a similar pattern to those of the experience sampling study [10]. One exception is the topic “chores”. The experience sampling study finds that people have low affect levels when doing chores, but in our study, the Facebook activity sentiment shows an opposite trend. Facebook sentiment *z*-score is 0.30, whereas experience sampling *z*-score is -0.21 (see Table 4). It is possible that people would like to discuss a chore on social media either before or after they carry out it, but not in the middle of the activity. For some people, the anticipation of getting things done may negate their apprehension of work and the sense of achievement after having completed a task may lead to an overall more positive profile. However, when people are asked how happy they are while doing the chores, as in the experience sampling method, they’ll very likely give a negative answer. Our results differ from those of the experience sampling study in that we measure overall impression of affect towards an experience, whereas that study measures affect during an experience. The Facebook status updates in our study cover more situations than the sampled experiences (see Fig 2). Our non-intrusive data analysis allows for a broader and potentially more detailed analysis than the intrusive moment-sampling approach.

**Table 4:** Facebook activity sentiment.

activities	FB sentiment (*z*-scores)	experience sampling (*z*-scores)
religion	0.57	
holiday	0.39	
talk to friend	0.39	0.35
chores	0.30	-0.21
family	0.27	
meal	0.29	0.19
school	-0.03	-0.21
maths	-0.15	-0.25

## Discussion

Our study explicitly illustrates how machine learning techniques quantify psychological richness in social media language. It has been shown that computer-based predictions utilizing social media data can reasonably predict SWL and general affect of users [13,16,17,21]. Combining both LIWC topics and machine generated topics yields the best result in our SWL prediction model. Integrating affect with machine-predicted SWL as a SWB profile is able to predict depression symptoms. These prediction results are clinically important, since SWL has been found to be associated with health outcomes [22,25,24], economics and historical events [20]. Using social media data may allow for less intrusive SWL sampling as well as repeated sampling in a way that avoids ‘adaptation to the test’. It also offers the option of real time sampling of psychological measures.

In our study, most of the features in the SWL prediction model are selected automatically by computer. LDA contributes most of the language features to the model. Considering that the choice of method in LDA depends on the number of documents and the LDA results generated from the 2612 documents show poor performance in predicting SWL (*r* = 0.13), we adopt a word count approach making use of an LDA term table from a larger study. Manual comparison of these features with manually created topic lists from the literature shows that machine selection indeed selects factors previously identified as important to SWL, including both internal (e.g., cognitive structure) and external ones (e.g., health). Therefore, automated, data-driven methods are also able to identify language features comparable to those found in traditional studies based on manually created topics.

Natural language processing studies often use sentiment analysis to investigate affect. However, most previous studies involve only the intensity of affect [39,16,17,18], whereas the frequency of affect, which is the basis of affective well-being [27], has often been ignored. Our results show that the frequency of negative affect has a higher correlation with SWL (*r* = -0.23, *p* < 0.01) than the intensity of affect (*r* = 0.21, *p* < 0.01), indicating that the frequency of affect is an important component in sentiment analysis.

There are a few factors that hinder us from achieving an even higher correlation between machine-predicted and self-reported SWL. First, Facebook users often have a large audience that they may or may not know in their real lives, so they tend to disclose less intimate or negative information to such an audience due to concerns of social desirability [48]. As shown in our sample, 2088 out of the 2612 users have more positive status than negative status. In addition, some factors that greatly contribute to SWL are hard to detect in language data. For example, moderate aspiration is critical to SWL; people with high aspirations in life are less likely to be satisfied with their lives, because they will be disappointed by the gap between what they want and the reality [3]. Due to these reasons, Facebook status updates may not accurately reflect people’s actual SWL levels all the time, especially when others can access users’ status updates. To achieve a greater correlation with self-reported value, we may combine the data from various social media accounts of a single user. For example, aspiration may be more evident in the career profiles like those in LinkedIn accounts.

In addition to the prediction models, we also analyze the valence when people mention different activities, objects or situations. The valence levels towards certain activities indicated by the Facebook language are similar to those found using an experience sampling approach. People tend to be happier or less so when engaged in different activities. The valence-based approach does not require participants to intentionally report how happy they are, as in the experience sampling study; instead, the affect level can be inferred from the valence words and smileys in their language. This approach is more objective than the experience sampling approach, because participants’ specific updates would converge into a more natural and less biased indicator of how they regularly feel, rather than how they might respond when prompted. In addition, in an experience sampling study, participants might become annoyed or suffer similar changes when interrupted to be asked how they feel, e.g. during a laborious activity or in an inappropriate place like a theatre or cinema. Such invasive nature of experience sampling may lead to biases.

## Conclusion

Our study illustrates several advantages of computer models over self-reported evaluation.

First, clinical studies often measure SWL as a criterion for well-being of a patient. However, another key construct of SWB, affect, is seldom addressed in investigation, because it is laborious to frequently monitor affect through repeated surveys. Participants in many previous laboratory and clinical studies need to compulsorily report their affect levels many times a day [10]. This would consume considerable time and be unable to measure affect of an individual in the long term, because the experiment usually lasts for at most a few months. By contrast, we adopt a more naturalistic and objective way to assess affect by analyzing the valence in participants’ Facebook status updates. Participants report their moods and life events out of their personal intensions.

Second, in longitudinal studies or assessments of intervention outcomes, repeated measurements using the same scale within a short period of time could be inaccurate, because patients may keep providing the same answers (false ‘reliability’). By contrast, the computer prediction model based on natural language from social media is more objective. Participants do not need to adapt their answers to overt repeated testing. In addition, computer-based models can capture subconscious clues like self-esteem or optimism, without being affected by temporary emotions or feelings.

Third, a computer model can access pre-existing longitudinal data with reduced dropout chance and enhanced ability of mapping the progression of pre-clinical pathology. Accumulation of digital footprints on the Internet enables computer models to conduct analyses according to different timelines and to generate results in various stages of an individual’s life. Participants simply login to the system with a social media account, and the result based on their ten (or more) years of digital footprints can be automatically generated within a few minutes.

Our study highlights the necessity of incorporating social media data in multivariate appraisals of psychological situations. Automatic differentiation of a large volume of social media data into domain specific sentiment, affect, and other attitudes adds decisive values to the usually short and survey-based methodology. Future work can extend this approach by constructing more comprehensive psychological profiles that consist of multiple machine-assessed components based on combinations of different social media outputs.

## Supporting information

S1 Text Investigaton of 30-day period immediately prior to SWL survey completion. Here we repeat the SWL pipeline for a limited time period (30 days) for each user. We also study the impact of two different thresholds for minimum number of status updates per user.
(DOCX)

S1 Table Topic and topic words for Facebook activities and the ratings from two raters. The list of words relating to activities used for activity sentiment analysis.
(DOCX)

S2 Table Variable importance table, ranked in descending order. The variables are ranked in descending order according to the mean decrease in accuracy.
(DOCX)

S1 Fig Variable importance graph. The graph shows the top 50 important topics in the random forest model.
(TIF)
