# Supplementary material for: Building a profile of subjective well-being for social media users
Source: PLoS One. 2017 Nov 14;12(11):e0187278. doi: 10.1371/journal.pone.0187278 (PMC5685571; doi:10.1371/journal.pone.0187278)
Supplement: S1 Text — Here we repeat the SWL pipeline for a limited time period (30 days) for each user. We also study the impact of two different thresholds for minimum number of status updates per user. (DOCX) [file pone.0187278.s001.docx]

**Supplementary Text: Investigation of 30-day period immediately prior to SWL survey completion**

We repeated the analysis for the 30-day period preceding completion of the SWL survey for each user. 449 users did not have any status updates within their 30-day period. The maximum number of posts was 304, but that user is the only one who had more than 200 posts. We investigate the content of those posts to ensure that user account is not a spam account and that user is simply an earnest user, probably of young age (mentions classmates a lot) making very frequent, short posts.

We choose users with 10+ posts (*n* = 1018) and those with 20+ posts (*n* = 476) to create two datasets for exploring the information contained in status updates from 30 days immediately prior to the SWL survey completion.

Following the same processing pipeline in the paper, we first look at the correlation between sentiment contents of posts and self-reported SWL, and then create random forest models to predict SWL using different combinations of features.  The R script is provided in the same GitHub repository as in the paper.

The sentiment correlation indicates that the frequency of negative words and the mean sentiment scores are significantly correlated with the self-reported SWL in both datasets (see Table S1). The dataset with 20+ posts produce better correlations for both metrics. The increase in data per user may improve the quality of information. Notably, the frequency of positive words does not perform well in either case (low correlation coefficient and borderline significance) and the correlation coefficient of 0.08 is similar to that observed in the full dataset (0.078). This supports the idea that the positive bias observable in Facebook posts might reduce useful information (with respect to SWL) within positive posts overall.

**Table S1.** Correlations (*r*) between sentiment words (positive frequency, negative frequency, mean sentiment) in the two datasets restricted to a 30-day period prior to SWL survey completion.

| **Minimum number of posts** | **Frequency of negative words** | | **Frequency of positive words** | | **Mean sentiment** | |
| --- | --- | --- | --- | --- | --- | --- |
|  | *r* | *p* value | *r* | *p* value | *r* | *p* value |
| 10 | -0.14 | 8.91e-06 | 0.08 | 0.01 | 0.13 | 3.63e-05 |
| 20 | -0.22 | 1.07e-06 | 0.08 | 0.07 | 0.18 | 4.74e-05 |

**Random Forest Models:**

The 'min 10 post' dataset is split into training (*n* = 700) and test (*n* = 318) datasets, and the 'min 20 post' dataset is split into training (*n* = 322) and test (*n* = 148) datasets.

Random forest models are built using the same procedure as the full dataset. Table S2 shows the general performance of predicting the test set for four combinations of features (LIWC only, LDA only, LDA + Sentiment metrics, LDA + LIWC + Sentiment metrics). Performance is measured using correlation coefficient (including unadjusted *p* value) and root mean squared error (RMSE). In each case, the 30-day data performs less well than the full dataset. That said, there are still significant correlations between the predictions and the observed values, indicating that these reduced subsets are still informative. There is a clear challenge in testing the benefits of more data per user (increasing the minimum number of posts) and having more data and more users overall for training the model (reducing the minimum number of posts). We can explore this further by choosing random subsets that vary in number of posts but are matched for ‘*n*’. However, regarding the purposes of this paper, it is sufficient to show that there is useful information within even relatively small numbers of posts from limited time windows.

Table S2. Performance of random forest models for data restricted to a 30-day period prior to the SWL survey completion. Performance metrics include correlation coefficient (*r*), *p* value for correlation and root mean squared error (RMSE).

|  | **Minimum number of posts** | ***r*** | ***p* value** | **RMSE** |
| --- | --- | --- | --- | --- |
| LIWC | 10  20 | 0.24  0.20 | 1.39e-05  0.013 | 1.33  1.42 |
| LDA | 10  20 | 0.15  0.14 | 0.008  0.09 | 1.36  1.44 |
| LDA + sentiment | 10  20 | 0.14  0.19 | 0.01  0.018 | 1.36  1.42 |
| LDA + LIWC + sentiment | 10  20 | 0.17  0.24 | 0.0014  0.003 | 1.35  1.41 |
