# Supplementary material for: Building a profile of subjective well-being for social media users
Source: PLoS One. 2017 Nov 14;12(11):e0187278. doi: 10.1371/journal.pone.0187278 (PMC5685571; doi:10.1371/journal.pone.0187278)
Supplement: S1 Table — The list of words relating to activities used for activity sentiment analysis. (DOCX) [file pone.0187278.s002.docx]

**Table S3.** Topic and topic words for Facebook activities and the ratings from two raters

| **Activity** | **Words** | **Rater 1** | **Rater 2** |
| --- | --- | --- | --- |
| talk with friends | chat & friend | 4 | 5 |
|  | talk & friend | 5 | 5 |
|  | speak & friend | 5 | 5 |
|  | tell & friend | 5 | 5 |
|  | told & friend | 4 | 5 |
| school | study | 5 | 5 |
|  | lecture | 5 | 4 |
|  | homework | 4 | 5 |
|  | assignment | 4 | 4 |
|  | paper | 3 | 4 |
|  | exam | 5 | 5 |
|  | research | 3 | 3 |
|  | science | 4 | 3 |
|  | literature review | 2 | 2 |
|  | quiz | 4 | 3 |
|  | course | 5 | 3 |
|  | graduate | 4 | 3 |
|  | academic | 4 | 4 |
|  | assessment | 3 | 4 |
|  | assay | 2 | 1 |
|  | report | 3 | 3 |
| mathematics | mathematics | 5 | 5 |
|  | maths | 5 | 5 |
|  | algebra | 4 | 5 |
|  | calculation | 5 | 5 |
|  | geometric | 4 | 5 |
|  | calculus | 4 | 5 |
| family | my child | 4 | 5 |
|  | my father | 5 | 5 |
|  | my mother | 5 | 5 |
|  | my husband | 5 | 5 |
|  | my wife | 5 | 5 |
|  | my kid | 4 | 5 |
|  | my aunt | 3 | 5 |
|  | my uncle | 3 | 5 |
|  | my brother | 4 | 5 |
|  | my sister | 4 | 5 |
|  | my cousin | 3 | 5 |
|  | my grandpa | 4 | 5 |
|  | my grandma | 4 | 5 |
|  | my grandad | 4 | 5 |
|  | mom | 4 | 5 |
|  | dad | 4 | 5 |
| holiday | vacation | 5 | 5 |
|  | holiday | 5 | 5 |
|  | weekend | 4 | 4 |
|  | Saturday | 4 | 3 |
|  | Sunday | 4 | 3 |
| religion | faith | 5 | 5 |
|  | church | 4 | 5 |
|  | pray | 4 | 5 |
|  | pastor | 4 | 5 |
|  | temple | 3 | 5 |
|  | monk | 3 | 5 |
|  | chapel | 4 | 5 |
|  | choir | 3 | 3 |
|  | spiritual | 3 | 4 |
|  | Buddhist | 3 | 5 |
| meal | meal | 5 | 5 |
|  | dinner | 4 | 5 |
|  | lunch | 4 | 5 |
|  | breakfast | 4 | 5 |
|  | food | 5 | 5 |
|  | dine | 4 | 5 |
|  | eat | 5 | 5 |
|  | cook | 4 | 4 |
|  | supper | 4 | 5 |
|  | snack | 4 | 5 |
|  | bread | 3 | 4 |
|  | cake | 3 | 4 |
|  | meat | 3 | 4 |
|  | pasta | 3 | 4 |
|  | rice | 3 | 4 |
|  | soup | 3 | 4 |
|  | steak | 3 | 4 |
|  | chicken | 3 | 4 |
|  | burger | 3 | 4 |
|  | sandwich | 3 | 4 |
| chores | chores | 5 | 5 |
|  | housework | 4 | 5 |
|  | laundry | 4 | 5 |
|  | clean | 4 | 4 |
|  | dishwashing | 4 | 5 |
|  | grocery | 3 | 4 |
|  | sweep | 4 | 5 |
|  | vacuum | 4 | 5 |
|  | mow | 3 | 5 |
| death, sick | sick | 4 | 5 |
|  | pain | 4 | 4 |
|  | ill | 4 | 5 |
|  | unwell | 4 | 5 |
|  | dizzy | 3 | 4 |
|  | puke | 3 | 5 |
|  | fever | 4 | 5 |
|  | vomit | 3 | 5 |
|  | die | 5 | 5 |
|  | cold | 4 | 3 |
