# Supplementary material for: Building a profile of subjective well-being for social media users
Source: PLoS One. 2017 Nov 14;12(11):e0187278. doi: 10.1371/journal.pone.0187278 (PMC5685571; doi:10.1371/journal.pone.0187278)
Supplement: S2 Table — The variables are ranked in descending order according to the mean decrease in accuracy. (DOCX) [file pone.0187278.s003.docx]

**Table S4.** Variable importance table, ranked in descending order.

| **Topic names** | **Mean decrease in accuracy** |
| --- | --- |
| neg.freq | 2.153132e-02 |
| pos.neg | 1.711143e-02 |
| mean.senti | 1.538975e-02 |
| topic812 | 4.403136e-03 |
| topic217 | 4.144345e-03 |
| topic1620 | 4.116363e-03 |
| topic1873 | 3.796942e-03 |
| topic1573 | 3.792032e-03 |
| topic48 | 3.554843e-03 |
| topic205 | 3.016386e-03 |
| topic253 | 2.655263e-03 |
| Topic51 | 2.511755e-03 |
| Topic38 | 2.471916e-03 |
| Topic1715 | 2.069146e-03 |
| Topic470 | 2.023357e-03 |
| Topic605 | 1.899427e-03 |
| Topic1038 | 1.700280e-03 |
| Topic111 | 1.639010e-03 |
| Topic815 | 1.626409e-03 |
| Topic1684 | 1.622926e-03 |
| Topic 126 | 1.506155e-03 |
| Topic 1980 | 1.452853e-03 |
| Topic 765 | 1.430752e-03 |
| Topic 321 | 1.398145e-03 |
| Topic 555 | 1.396049e-03 |
| Topic 259 | 1.370874e-03 |
| Topic 939 | 1.365104e-03 |
| Topic 171 | 1.161924e-03 |
| Topic 379 | 1.160562e-03 |
| Topic 754 | 1.041007e-03 |
| Topic 994 | 1.036600e-03 |
| Topic 1725 | 8.562196e-04 |
| Topic 15 | 7.558967e-04 |
| Topic 327 | 6.810455e-04 |
| Topic 855 | 6.477440e-04 |
| Topic 638 | 6.390853e-04 |
| Topic 1440 | 6.234051e-04 |
| Topic 700 | 5.336496e-04 |
| Topic 1530 | 3.969067e-04 |
| Topic 505 | 3.645587e-04 |
| Topic 314 | 2.948439e-04 |
| Topic 846 | 2.630950e-04 |
| Topic 1411 | 2.618081e-04 |
| Topic 1316 | 2.512987e-04 |
| Topic 1179 | 1.911284e-04 |
| Topic 1808 | 1.806405e-04 |
| Topic 1221 | 4.421061e-05 |
| Topic 675 | -1.001184e-04 |
| Topic 1819 | -1.006158e-04 |
| Topic 588 | - 1.046427e-04 |
