# Supplementary figures and images for: Building a profile of subjective well-being for social media users
Source: PLoS One. 2017 Nov 14;12(11):e0187278. doi: 10.1371/journal.pone.0187278 (PMC5685571; doi:10.1371/journal.pone.0187278)

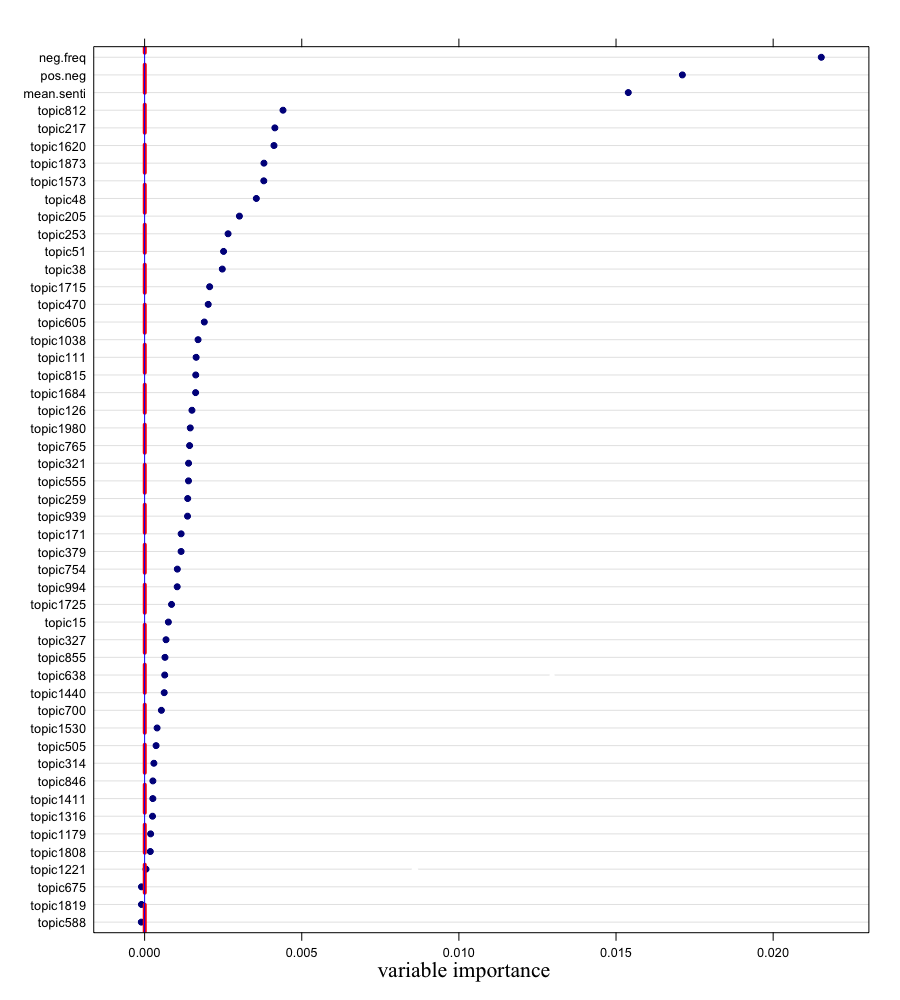

Supplement: S1 Fig — The graph shows the top 50 important topics in the random forest model. (TIF) [file pone.0187278.s004.tif]
